# K34 (3,3″-Dihydroxy-6′-desmethyl Terphenyllin) Promotes Hemoglobin-Supported H_2_O_2_ Decomposition and Attenuates Neurodegenerative Phenotypes in APP/PS1 Mice with Focal GiD

**DOI:** 10.3390/antiox15101293

**Published:** 2026-10-05

**Authors:** Yongmin Mason Park, Byeoung-Kyu Choi, Mingu Gordon Park, Myeongju Kim, Woojin Won, Hee Jae Shin, C. Justin Lee

**Affiliations:** 1 Center for Memory and Glioscience, Institute for Basic Science, Daejeon 34126, Republic of Korea; dydalsqkr94@ibs.re.kr (Y.M.P.); phm0090@g.skku.edu (M.K.); wwj@ibs.re.kr (W.W.); 2 IBS School, University of Science and Technology (UST), Daejeon 34113, Republic of Korea; 3 Department of Bio-convergence Engineering, Dongyang Mirae University, Seoul 08221, Republic of Korea; choibk4404@dongyang.ac.kr; 4 Department of Biomedical Engineering, Sungkyunkwan University, Suwon 16419, Republic of Korea; 5 Marine Natural Products Chemistry Laboratory, Korea Institute of Ocean Science and Technology, Busan 49111, Republic of Korea; 6 Department of Marine Biotechnology, University of Science and Technology (UST), Daejeon 34113, Republic of Korea

**Keywords:** hydrogen peroxide, hemoglobin, pseudoperoxidase, Alzheimer’s disease, oxidative stress, marine natural product

## Abstract

Hydrogen peroxide (H_2_O_2_) contributes to oxidative stress in Alzheimer’s disease (AD), yet conventional antioxidants that directly scavenge H_2_O_2_ have shown limited therapeutic efficacy. Here, we investigated K34 (3,3″-dihydroxy-6′-desmethyl terphenyllin), a catechol-bearing para-terphenyl isolated from the marine fungus *Penicillium janthinellum* 168CLC-17.1, as an indirect H_2_O_2_-decomposing agent. Complementary cell-free assays showed that K34 promoted peroxidase-dependent H_2_O_2_ decomposition, including Hb-supported H_2_O_2_ decomposition, while molecular docking predicted a plausible binding pose near the Hbβ heme pocket. In amyloid-β_42_ (Aβ_42_)-stimulated primary astrocytes, K34 reduced intracellular H_2_O_2_. In APPswe/PSEN1dE9 (APP/PS1) mice subjected to focal GFAP-inducible diphtheria toxin receptor (fGiD), systemic K34 attenuated memory impairment, preserved NeuN-positive neurons, reduced GFAP immunoreactivity, and improved hippocampal spike probability. K34 also exhibited low passive blood–brain barrier (BBB) permeability and limited inhibition of five major cytochrome P450 (CYP) isoforms under the tested conditions. These findings extend Hb-supported H_2_O_2_ decomposition to a structurally distinct marine natural product and support further evaluation of K34 as an antioxidant scaffold for modulating oxidative stress-associated neurodegenerative phenotypes.

## 1. Introduction

Hydrogen peroxide (H_2_O_2_) occupies a paradoxical position in central nervous system physiology, functioning both as a tightly regulated second messenger in redox signaling and, when its production becomes excessive, as a cytotoxic driver of oxidative damage [1,2]. In the brain, the loss of redox homeostasis is increasingly recognized as an important contributing mechanism in neurodegenerative diseases, including Alzheimer’s disease (AD), where oxidative modification of proteins and nucleic acids impairs synaptic function and precipitates neuronal loss [1]. Among the cellular sources of pathological H_2_O_2_, reactive astrocytes have emerged as key players: upon activation, they generate aberrant levels of H_2_O_2_ that propagate oxidative stress and directly contribute to neurodegeneration in AD [3,4]. These findings provide a rationale for investigating whether selective reduction of pathological H_2_O_2_ can mitigate oxidative-stress-associated neurodegenerative phenotypes.

Efforts to counteract oxidative stress have historically relied on direct antioxidants and radical scavengers. However, agents such as sodium pyruvate and N-acetylcysteine that directly consume H_2_O_2_ typically require high doses to be effective and carry the risk of nonspecific reactivity or prooxidant effects. At the same time, indiscriminate scavenging may disrupt the physiological signaling roles of H_2_O_2_ [5,6]. Dietary antioxidants such as curcumin and resveratrol have similarly faltered in clinical development owing to inconsistent efficacy and poorly defined mechanisms [5]. These limitations highlight the need for approaches that indirectly and in a controlled manner reduce pathological H_2_O_2_, rather than through direct consumption.

An alternative and mechanistically distinct strategy has recently emerged from the recognition that hemoglobin (Hb), a heme-containing protein long regarded primarily as an oxygen carrier, is also expressed in the brain, including in astrocytes [7,8,9]. Hb plays a context-dependent, dual role in redox biology: extracellular or oxidatively modified Hb can behave as a Fenton reagent that propagates oxidative damage, whereas endogenous Hb possesses an intrinsic pseudoperoxidase activity capable of decomposing H_2_O_2_ [10,11]. Under physiological conditions this pseudoperoxidase activity is weak and provides only modest antioxidative protection. Recently, however, we established that this latent activity can be harnessed pharmacologically: synthetic small molecules such as KDS12025, built on an N-phenethylaniline scaffold bearing an electron-donating amine group, markedly enhance Hb pseudoperoxidase activity and enable efficient H_2_O_2_ decomposition even at low Hb levels, without compromising oxygen transport [4]. Such peroxidase-enhancing compounds therefore constitute a distinct class of antioxidant that amplifies an endogenous detoxification pathway rather than directly scavenging H_2_O_2_. Here, we asked whether a structurally distinct, naturally occurring scaffold could promote Hb-supported H_2_O_2_ decomposition in a purified-protein system.

Marine-derived microorganisms are a rich and underexplored source of structurally diverse small molecules with therapeutic potential [12,13,14]. Among these scaffolds, the p-terphenyls are a distinctive family of fungal aromatic metabolites: terphenyllin, the parent compound, was originally isolated from *Aspergillus candidus* [15] and related p-terphenyls have since been reported across diverse fungi to exhibit a range of bioactivities, including antibacterial, cytotoxic and antitumor, anti-inflammatory, and α-glucosidase–inhibitory effects [16,17]. More broadly, naturally occurring catechol-containing polyphenols, including epigallocatechin gallate (EGCG), quercetin, and piceatannol, have been investigated for neuroprotective effects in AD-related experimental models through diverse antioxidant, anti-inflammatory, and amyloid-related mechanisms [18,19,20,21]. Despite the established redox activity of such natural polyphenols, whether a structurally distinct fungal p-terphenyl can promote H_2_O_2_ decomposition through an enzyme-dependent, indirect mechanism remains unclear. In the course of screening secondary metabolites from marine fungi, we previously reported the isolation of five compounds, including the para-terphenyl K34, and evaluated their anti-neuroinflammatory activity in lipopolysaccharide-stimulated microglial cells [13]. In that screen, K34 showed only weak activity accompanied by cytotoxicity and was not pursued further. The same compound had also been isolated from *Penicillium chermesinum* and evaluated in an earlier acetylcholinesterase-inhibition study, where it showed no detectable activity [22]. However, neither these previous studies nor our earlier work examined its H_2_O_2_-related redox pharmacology or whether it could act through an enzyme-dependent mechanism. As a para-terphenyl, K34 is structurally distinct from the synthetic N-phenethylaniline scaffold characterized in our previous study, making it an informative natural-product scaffold for examining Hb-supported H_2_O_2_ decomposition. Clarifying the H_2_O_2_-decomposing mechanism of K34 is therefore relevant not only to the pharmacology of this compound but also to evaluating whether this mechanism could attenuate oxidative-stress-associated neurodegenerative phenotypes.

In this study, we investigated K34, isolated and purified from marine-derived microorganisms, as a candidate H_2_O_2_-decomposing agent. Combining cell-free assays, molecular docking, amyloid-β (Aβ)-stimulated primary astrocytes, and APP/PS1-based fGiD AD model [3], we asked whether K34 reduces pathological H_2_O_2_ and, if so, whether it acts through direct H_2_O_2_ consumption or through peroxidase-dependent H_2_O_2_ decomposition. We further examined whether Hb supports this activity in a purified-protein system. 

## 2. Materials and Methods

### 2.1. Isolation and Purification of K34

K34 was isolated from the culture extract of the marine-derived fungus *Penicillium janthinellum* 168CLC-17.1 as previously described [13]. The fungal strain was isolated from a marine sediment sample collected from Cu Lao Cham Island, Vietnam, and identified based on morphological characteristics and internal transcribed spacer (ITS) ribosomal DNA (rDNA) sequence analysis. The strain was cultivated on a rice medium prepared with natural seawater under static conditions at 28 °C for 22 days. The fermented cultures were extracted with ethyl acetate (EtOAc), and the resulting crude extract was fractionated by ODS flash chromatography, followed by repeated octadecylsilyl (ODS) column chromatography and semi-preparative reversed-phase high-performance liquid chromatography (HPLC) to afford pure K34. The structure of K34 was elucidated by analysis of negative-ion electrospray ionization mass spectrometry (ESIMS) together with 1D (^1^H and ^13^C) and 2D (Heteronuclear Single Quantum Coherence (HSQC) and Heteronuclear Multiple Bond Correlation (HMBC)) nuclear magnetic resonance (NMR) spectroscopic data.

### 2.2. In Vitro H_2_O_2_ Decomposition Assays

K34 was dissolved in dimethyl sulfoxide (DMSO) and serially diluted to the indicated final concentrations. Hydrogen peroxide (H_2_O_2_; Cat# H1009, Sigma-Aldrich, St. Louis, MO, USA), horseradish peroxidase (HRP; Cat# P8125, Sigma-Aldrich, St. Louis, MO, USA), hemoglobin (Hb; Cat# H7379, Sigma-Aldrich, St. Louis, MO, USA), and Amplex Red reagent (Cat# A12222, Invitrogen, Waltham, MA, USA) were used as supplied.

**Amplex Red assay.** H_2_O_2_ (4 μM final concentration) was prepared in 50 mM sodium phosphate buffer (pH 7.2). A 49 μL aliquot of this solution was combined with 1 μL of K34 or vehicle (DMSO), then supplemented with an assay mixture containing Amplex Red (0.1 mM) and either HRP (0.2 U/mL) or Hb (10 μg/mL) as the peroxidase enzyme. Reactions were dispensed into 96-well plates at 100 μL per well and incubated at 37 °C for 60 min. Resorufin fluorescence was measured at excitation/emission wavelengths of 535/580 nm using a SpectraMax iD5 microplate fluorescence reader (Molecular Devices, San Jose, CA, USA). Residual H_2_O_2_ was calculated using the following equation: (sample fluorescence − blank fluorescence)/(control fluorescence − blank fluorescence) × 100.

**ROS-Glo assay.** H_2_O_2_ (10 μM) and K34 were prepared as described above, with HRP (0.2 U/mL) or Hb (10 μg/mL) added as the peroxidase enzyme where indicated. The reaction mixture was dispensed into each well at 100 μL and incubated at 37 °C for 60 min. The ROS-Glo H_2_O_2_ substrate solution (Cat# G8820, Promega, Madison, WI, USA) was then added and incubated at room temperature for 30 min, after which the ROS-Glo detection solution was added and incubated for a further 15 min. Luminescence was recorded using the same SpectraMax iD5 microplate reader. Residual H_2_O_2_ level (% of control) was calculated as: (sample luminescence − blank luminescence)/(control luminescence − blank luminescence) × 100.

For experiments comparing HRP and Hb as peroxidase enzymes, and for direct comparison with KDS12025, identical assay conditions were applied in parallel across both platforms. All cell-free assays were performed in triplicate wells per concentration, and each dose–response curve was fitted to the pooled triplicate data. Dose–response curves and half-maximal effective concentration (EC_50_) values were determined by fitting the data to a four-parameter logistic model in GraphPad Prism (ver. 10.3.1; GraphPad Software, Boston, MA, USA) using least-squares regression with 95% confidence intervals.

### 2.3. Molecular Docking and Binding Energy Calculation

The chemical structures of K34 and KDS12025 were generated and ionized at pH 7.4 ± 1.0 using the Epik ionizer and energy-minimized with the OPLS_2005 force field using the LigPrep tool in Maestro (ver. 14.2, Schrödinger LLC, New York, NY, USA). The X-ray crystal structure of human oxyhemoglobin (PDB: 2DN1) was retrieved from the Protein Data Bank and prepared using the Protein Preparation Workflow in Maestro at physiological pH (7.4 ± 1.0). Hydrogen atoms and missing side chains were added, water molecules within 5 Å of the heme protein in the Hbβ subunit were removed, and the structure was energy-minimized with the OPLS_2005 force field. To mimic the catalytically relevant ferryl intermediate of the Hb pseudoperoxidase cycle, the heme iron of the Hbβ subunit was set to the Fe(IV)=O (Compound II) oxidation state. A hydrogen peroxide molecule was placed within the Hbβ heme-binding pocket proximal to the oxoiron and the distal histidine (HIS-63), consistent with the proposed pseudoperoxidase reaction geometry. A docking grid of 36 Å × 36 Å × 36 Å was centered on the heme-binding pocket of the Hbβ subunit. Docking was performed using the Glide module in standard precision (SP) mode and poses with positive Coulombic or van der Waals energies were excluded. The top-scoring pose for each ligand was selected and subjected to binding free energy estimation using the molecular mechanics/generalized Born surface area (MM-GBSA) method (ΔG_bind_ = G_Complex_ − G_Protein_ − G_Ligand_) implemented in the Prime module with the OPLS_2005 force field and VSGB 2.0 solvation model. Key interacting residues and interaction types—including hydrogen bonds, π–π stacking, and π–cation interactions—as well as distances between H_2_O_2_ and the ligand and protein, were identified and visualized for each ligand. Because docking protocols and scoring functions differ across software platforms, binding energies reported in this study are not directly comparable to ΔG_bind_ values obtained using other docking software.

### 2.4. Primary Astrocyte Culture

Primary hippocampal astrocytes were prepared from postnatal day 0–2 C57BL/6 mouse pups. Hippocampal tissue was dissected, freed of meninges, minced, and dissociated into a single-cell suspension by mechanical trituration. Cells were cultured in Dulbecco’s modified Eagle’s medium (Cat# 10013153, Corning, Corning, NY, USA) supplemented with D-glucose (4500 mg/L), L-glutamine, sodium pyruvate (110 mg/L), 10% heat-inactivated horse serum, 10% heat-inactivated fetal bovine serum, and 1000 U/mL penicillin–streptomycin, and maintained at 37 °C in a humidified 5% CO_2_ incubator as previously described [23].

### 2.5. Preparation of Oligomerized Amyloid-β_42_

Amyloid-β42 (Aβ_42_; Cat# RP10017-1, GenScript, Piscataway, NJ, USA) monomers were dissolved in 1% ammonium hydroxide to a stock concentration of 1 mM and further diluted in phosphate-buffered saline (PBS). The diluted solution was incubated at 4 °C for 24 h to allow oligomerization, then stored at −80 °C until use as previously described [4].

### 2.6. Intracellular ROS/H_2_O_2_ Detection and WST-8 Assay in Astrocytes

Two complementary methods were used to assess intracellular H_2_O_2_ levels in Aβ42-stimulated primary astrocytes as previously described [4].

**DCFDA assay.** Astrocytes were seeded in black 96-well plates (Cat# 89626, ibidi, Gräfelfing, Germany) and treated with oligomerized Aβ_42_ (5 μM) in the presence or absence of K34. Cells were subsequently loaded with 2′,7′-dichlorodihydrofluorescein diacetate (DCFDA; Cat# D399, Invitrogen, Waltham, MA, USA) and incubated at 37 °C in the dark, and fluorescence intensity was measured at 485/530 nm (excitation/emission) using a microplate reader; values were normalized to the vehicle control after background subtraction. All DCFDA measurements were performed in triplicate wells per condition.

**Real-time oROS-G imaging.** Primary cultured astrocytes were transfected with the adeno-associated virus serotype 5 (AAV5)-GFAP104-oROS-GFP-4X6T viral vector on DIV11 and seeded into glass-bottom 96-well plates (Cat# 89627, ibidi, Gräfelfing, Germany). Three days later, cells were treated with oligomerized Aβ_42_ (5 μM) in the presence or absence of K34, sodium pyruvate or vehicle. Confocal live-cell imaging was performed using a A1R confocal microscope (Nikon, Tokyo, Japan) mounted on a Nikon Eclipse Ti body with a 20× objective lens. The plate was maintained in a live-cell imaging chamber at 37 °C and 10% CO_2_ for continuous recording over 40 h. For each condition, three independent wells were imaged, with four fields of view acquired per well. Fluorescence was quantified at the single-cell level, and each cell was treated as an individual data point (n); the number of cells analyzed therefore differed between conditions and is reported in the corresponding figure legend. Images were acquired and analyzed using NIS-Elements AR software (ver. 6.10; Nikon, Tokyo, Japan). Fitting endpoint fluorescence data determined EC_50_ values for the K34-mediated reduction in Aβ_42_-induced oROS-G fluorescence to a four-parameter logistic curve in GraphPad Prism (ver. 10.3.1).

**WST-8 assay.** To assess whether K34-associated reductions in intracellular ROS/H_2_O_2_ signals could be influenced by changes in cellular viability-associated metabolic activity, primary astrocytes were treated with K34 in the presence or absence of oligomerized Aβ_42_ (5 μM) for 40 h, under conditions corresponding to the cellular ROS/H_2_O_2_ experiments. Following treatment, viability-associated metabolic activity was assessed using a WST-8 assay (Cat# QM5000, BIOMAX, Guri, Republic of Korea) according to the manufacturer’s instructions. Treatment medium was replaced with fresh astrocyte culture medium, WST-8 reagent was added at 10% (*v*/*v*), and cells were incubated at 37 °C in 5% CO_2_ for 2 h. Absorbance was measured at 450 nm using a SpectraMax iD5 microplate reader. WST-8 signals were normalized to the vehicle-treated control for K34-alone conditions and to the Aβ_42_-treated control for Aβ_42_+K34 conditions.

### 2.7. Animal Husbandry

Experiments were performed on male and female C57BL/6 (B6) mice obtained from the Institute for Basic Science Animal Facility, or on APPswe/PSEN1dE9 (APP/PS1) mice on a B6C3 hybrid background (RRID: MMRRC_034829-JAX), originally obtained from Jackson Laboratory (stock no. 004462) and maintained as hemizygotes by crossing with B6C3 F1 mice. Genotyping of APP/PS1 mice was performed by PCR using the primers APP/PS1_F (5′-AATAGAGAACGGCAGGAGCA-3′) and APP/PS1_R (5′-GCCATGAGGGCACTAATCAT-3′). All mice were housed under a 12-h light/dark cycle (lights off at 20:00 h) with ad libitum access to food and water. All animal experiments were conducted in accordance with the ARRIVE 2.0 guidelines and approved by the Institutional Animal Care and Use Committee of IBS (Daejeon, Republic of Korea; IBS-2023-006; approved on 20 February 2024). B6 littermates (8 weeks old) were used for virus injection, followed by slice electrophysiology at 14 weeks of age. For the fGiD model, virus injection was performed on 5-month-old APP/PS1 mice and age-matched wildtype littermates.

### 2.8. Virus Injection and the APP/PS1-Based fGiD AD Model

Mice were anesthetized with isoflurane and head-fixed in a stereotaxic frame (RWD Life Science, Shenzhen, China). Following scalp incision, a burr hole was drilled above the hippocampus, and viruses (AAV5-gfaABC1D-GFP-4X6T or AAV5-gfaABC1D-DTR-GFP-4X6T, produced at the IBS Virus Facility) were bilaterally injected into the dentate gyrus (A/P −1.8 mm, D/V −1.9 mm from bregma, M/L ±1.2 mm from the skull surface), delivered via a stainless-steel injection needle at a rate of 0.1 μL/min for 10 min (1 μL per hemisphere). Three weeks after viral injection, diphtheria toxin (DT; Cat# D0564, Sigma-Aldrich, St. Louis, MO, USA) was administered intraperitoneally at 12 μg/kg/day once daily for 16 consecutive days to induce astrocyte-targeted ablation (fGiD model) as previously described [3], with concurrent intraperitoneal administration of K34 (10 mg/kg/day) or vehicle over the same period. The 10 mg/kg/day dose was selected empirically as an exploratory proof-of-concept dose for the in vivo efficacy experiment and was not based on pharmacokinetic exposure matching or formal dose optimization. Mice were assigned to vehicle or K34 treatment groups by simple randomization using a random number generator. The control + vehicle (Ctrl+Veh.), fGiD+Veh., and fGiD+K34 groups comprised 7/6, 7/10, and 9/5 female/male mice, respectively. No formal a priori sample size calculation was performed; group sizes were determined based on colony availability and were consistent with those used in related survival studies of this model. Survival was monitored daily throughout the DT administration period and for two additional days thereafter. Behavioral testing, electrophysiology, and immunohistochemistry were performed beginning on day 23 after the start of DT administration. One mouse exhibiting overt dermatological disease before DT injection was excluded from analysis.

### 2.9. Acute Brain Slicing

Brain slices were prepared from 6- to 7-month-old APP/PS1+fGiD mice and age-matched wildtype littermates as previously described [24]. Mice were deeply anesthetized with isoflurane, and brains were rapidly excised into ice-cold high-sucrose artificial cerebrospinal fluid (aCSF) containing (in mM): 212.5 sucrose, 26 NaHCO_3_, 10 D-(+)-glucose, 5 MgCl_2_, 3 KCl, 0.1 CaCl_2_, and 1.25 NaH_2_PO_4_. Coronal hippocampal slices (300 μm) were prepared using a vibrating microtome D.S.K LinearSlicer PRO7, Dosaka EM Co., Ltd., Kyoto, Japan) and allowed to recover for at least 1 h at room temperature in extracellular aCSF containing (in mM): 124 NaCl, 3 KCl, 24 NaHCO_3_, 2 CaCl_2_, 1.25 NaH_2_PO_4_, 1 MgCl_2_, and 10 D-(+)-glucose (310 mOsm/kg, pH 7.4). All solutions were continuously bubbled with 95% O_2_/5% CO_2_.

### 2.10. Evoked Spike Probability Recordings

Evoked spike probability was assessed in the same hippocampal slices described above, following a previously established protocol [25]. A bipolar tungsten electrode was positioned in the outer half of the middle third of the molecular layer of the dentate gyrus to stimulate lateral perforant path fibers (100-μs pulse duration) at 0.1 Hz, with stimulus intensities ranging from 100 to 1000 μA. Evoked excitatory postsynaptic potentials (EPSPs) were recorded using glass micropipette electrodes filled with an internal solution containing (in mM): 120 potassium gluconate, 10 KCl, 1 MgCl_2_, 0.5 EGTA, and 40 HEPES (pH 7.2). Spike probability was calculated as the ratio of the number of stimulations that generated a spike to the total number of stimulations delivered at each intensity. Evoked spike probability recordings were obtained from 9 hippocampal slices per group, derived from Ctrl+Veh. (N = 4; 2 female, 2 male), fGiD+Veh. (N = 5; 1 female, 4 male), and fGiD+K34 (N = 5; 3 female, 2 male) mice.

### 2.11. Passive Avoidance Test

Memory function was assessed using a two-compartment passive avoidance apparatus consisting of light and dark chambers separated by a sliding door (Ugo Basile, Gemonio, Italy). During the acquisition trial, mice were placed in the light compartment and allowed to explore for 60 s, after which the door was raised; upon entry into the dark compartment, the door was closed and a brief electric foot shock (0.5 mA, 2 s duration) was delivered through the grid floor. Twenty-four hours later, mice underwent a retrieval trial under identical conditions without shock delivery, and the latency to enter the dark compartment was recorded as an index of memory retention.

### 2.12. Immunohistochemistry and Image Quantification

Mice were anesthetized with isoflurane and transcardially perfused with 0.9% saline followed by ice-cold 4% paraformaldehyde (PFA) in 0.1 M PBS. Brains were post-fixed in 4% PFA at 4 °C overnight and cryoprotected in 30% sucrose for 48 h. Coronal sections (30 μm) were cut on a cryostat (Cat# CM1950, Leica, Wetzlar, Germany) and stored in glycerol-based storage solution at 4 °C until use. Sections were washed three times in 0.1 M PBS and blocked for 1 h in blocking solution (4% donkey serum, 0.3% Triton X-100 in 0.1 M PBS). Primary antibodies were applied in blocking solution and incubated overnight at 4 °C with gentle agitation. After three washes in 0.1 M PBS, sections were incubated with fluorescence-conjugated secondary antibodies in blocking solution for 2 h at room temperature. Following three additional washes—the first containing 4′,6-diamidino-2-phenylindole (DAPI, 1:1000) for nuclear counterstaining—sections were mounted using fluorescence mounting medium (Cat# S3023, Dako, Carpinteria, CA, USA) and air-dried as previously described [26].

Primary antibodies: guinea pig anti-neuronal nuclei (NeuN, 1:500, Cat# ABN90, Abcam, Cambridge, UK), chicken anti-glial fibrillary acidic protein (GFAP, 1:500, Cat# AB5541, Millipore, Burlington, MA, USA). Secondary antibodies: Alexa 488-conjugated donkey anti-chicken IgG (1:200, Cat# 703-545-155, Jackson ImmunoResearch, West Grove, PA, USA), Alexa 594-conjugated donkey anti-guinea pig IgG (1:200, Cat# 706-585-148, Jackson ImmunoResearch, West Grove, PA, USA). Images were acquired using a confocal microscope LSM900 (Zeiss, Jena, Germany). Z-stacked images (22–24 μm in 2 μm steps) were processed using ZEN Digital Imaging for Light Microscopy Blue software (Zeiss, ver. 3.2) and ImageJ (NIH, ver. 1.54b). GFAP-positive area was quantified from maximal-projection z-stack images using ImageJ. NeuN-positive CA1 pyramidal neurons were counted within a fixed 50 × 50 μm^2^ region of interest per section.

### 2.13. PAMPA-BBB Permeability and CYP Inhibition Assays

Both assays were performed by the Drug Development Support Center, Daegu Gyeongbuk Medical Innovation Foundation (DGMIF; Daegu, Republic of Korea).

**Parallel artificial membrane permeability assay for the blood–brain barrier (PAMPA-BBB).** Blood–brain barrier (BBB) permeability of K34 was assessed using the BBB PAMPA Explorer Test System (Pion Inc., Billerica, MA, USA) following a previously described method [27]. K34 was tested at 50 μM (pH 7.4, 25 °C) in a 96-well stirwell PAMPA sandwich (Cat# 110243, Pion Inc.) coated with BBB-1-lipid (Cat# 110672, Pion Inc.). Following 4 h of incubation, absorbance of donor and acceptor solutions was measured by UV detection (250–500 nm, 4-nm intervals) using a SYNERGY H1 microplate reader (BioTek, Winooski, VT, USA), and effective permeability (P_e_, × 10^−^^6^ cm/s) was calculated using PAMPA Explorer software (ver. 3.8, Pion Inc.) from triplicate measurements (mean ± SD). Compounds were classified as CNS+ (P_e_ > 10 × 10^−^^6^ cm/s) or CNS− (P_e_ < 10 × 10^−^^6^ cm/s). Progesterone (50 μM; Cat# P8783, Sigma-Aldrich, St. Louis, MO, USA) and ranitidine hydrochloride (50 μM; Cat# R101, Sigma-Aldrich) were included as central nervous system positive (CNS+) and negative (CNS−) reference compounds, respectively.

**CYP inhibition assay.** Inhibitory activity of K34 against five major human cytochrome P450 isozymes (CYP1A2, CYP2C9, CYP2C19, CYP2D6, and CYP3A4) was evaluated using a cocktail substrate assay with human liver microsomes (0.25 mg/mL; Cat# 452117, Corning, Corning, NY, USA) in 0.1 M potassium phosphate buffer (pH 7.4; Cat# 451201, Corning). K34 was tested at 0, 0.1, 0.5, 2, and 10 μM alongside isozyme-specific substrate probes: phenacetin (50 μM; CYP1A2), diclofenac (10 μM; CYP2C9), S-mephenytoin (100 μM; CYP2C19), dextromethorphan (5 μM; CYP2D6), and midazolam (2.5 μM; CYP3A4). Following pre-incubation at 37 °C for 5 min, reactions were initiated by addition of an NADPH regeneration system (Cat# V9510, Promega, Madison, WI, USA) and incubated at 37 °C for 15 min. Reactions were terminated by addition of acetonitrile containing terfenadine as an internal standard (Cat# T9562, Sigma-Aldrich), followed by centrifugation (15,000 rpm, 5 min, 4 °C). Metabolite formation was quantified by liquid chromatography–tandem mass spectrometry (LC–MS/MS) using a Nexera XR system (Shimadzu, Kyoto, Japan) coupled to a TSQ Vantage mass spectrometer (Thermo Fisher Scientific, Waltham, MA, USA) with a Kinetex C18 column (2.1 × 100 mm, 2.6 μm), Phenomenex, Torrance, CA, USA). Half-maximal inhibitory concentration (IC_50_) values were calculated using an inhibitory effect model in Phoenix WinNonlin (ver. 6.4; Pharsight, Certara, Princeton, NJ, USA) from duplicate experiments and classified as potent (IC_50_ < 1 μM), moderate (1–10 μM), or weak/absent (>10 μM). Ketoconazole (0.1 μM; Cat# K1003, Sigma-Aldrich) was included as a CYP3A4 reference inhibitor.

### 2.14. Quantification and Statistical Analysis

All quantification was performed in a blinded manner with respect to the experimental group. Sample sizes (n, individual cells, wells, or recordings; N, individual animals) are indicated in the corresponding figure legends. For single-cell oROS-G measurements, n denotes individual cells and N denotes the number of wells imaged per condition. Dose–response curves and EC_50_ values were generated using GraphPad Prism (ver. 10.3.1). Group comparisons for endpoint oROS-G fluorescence, passive avoidance performance, GFAP-positive area, and NeuN-positive cell counts were performed by one-way ANOVA followed by Tukey’s multiple comparison test. Spike probability at 600 μA was analyzed using the Kruskal–Wallis test. Survival was analyzed using the log-rank (Mantel–Cox) test. To evaluate the influence of sex on survival, Cox proportional hazards regression was additionally performed with treatment group and sex as covariates. Statistical significance was set at *p* < 0.05.

### 2.15. Use of Generative Artificial Intelligence

During the preparation of this manuscript, the authors used Claude (Opus 5; Anthropic, San Francisco, CA, USA) and ChatGPT (GPT 5.6 sol; OpenAI, San Francisco, CA, USA) for language refinement, including grammar, wording, and sentence-level clarity. The tool was not used for study design, data generation, data analysis, interpretation of results, or manuscript organization. All scientific content and conclusions were developed and verified by the authors.

## 3. Results

### 3.1. Isolation and Structural Identification of K34

**Figure 1:**
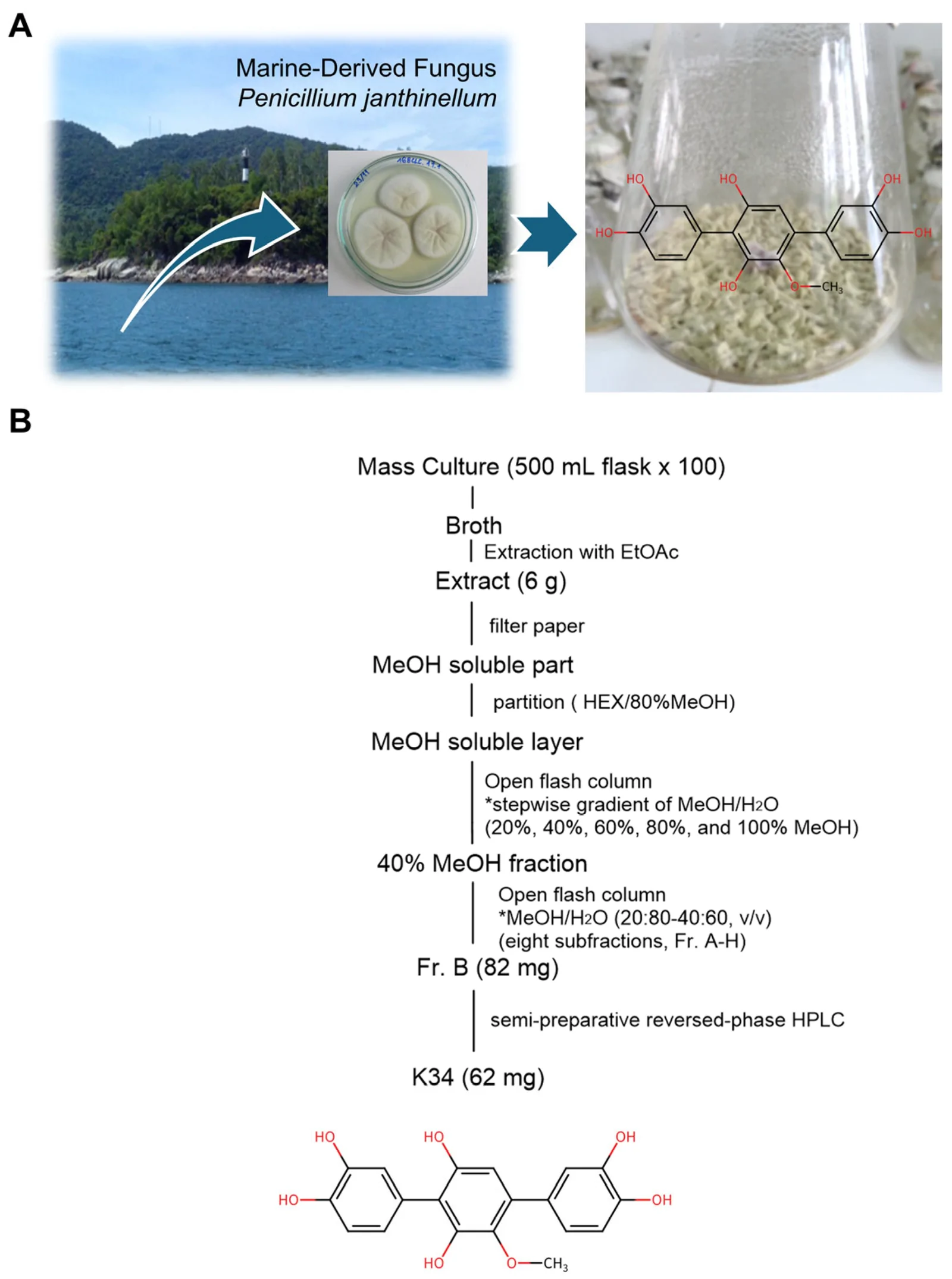
Isolation and purification of K34 from the marine-derived fungus *Penicillium janthinellum* 168CLC-17.1. (**A**) Isolation and cultivation of the K34-producing marine-derived fungus *Penicillium janthinellum* 168CLC-17.1 and the chemical structure of K34. (**B**) Isolation and purification scheme of K34. The asterisks (*) in (B) indicate that open flash column chromatography was performed using the specified solvent systems; the MeOH/H_2_O ratios denote the stepwise gradient or solvent compositions used for fractionation.

K34 was successfully isolated from cultures of the marine-derived fungus *Penicillium janthinellum* 168CLC-17.1 (Figure 1A). After large-scale cultivation, the EtOAc extract was sequentially purified by solvent partitioning, ODS flash chromatography, repeated ODS column chromatography, and semi-preparative reversed-phase HPLC to yield 62 mg of pure K34 (Figure 1B). The chemical structure of K34 was established by comprehensive spectroscopic analyses, including negative-ion ESIMS and 1D (^1^H and ^13^C) and 2D (HSQC and HMBC) NMR experiments. The negative-ion ESIMS spectrum exhibited a molecular ion peak at *m*/*z* 355.04 [M − H]−, consistent with the molecular formula of K34. Furthermore, the ^1^H, ^13^C, HSQC, and HMBC spectra confirmed the characteristic substitution pattern of the para-terphenyl skeleton. Comparison of these spectroscopic data with previously reported values identified K34 as 3,3″-dihydroxy-6′-desmethyl terphenyllin. Representative spectroscopic data are provided in the Supporting Information (Figure S1).

### 3.2. K34 Decomposes H_2_O_2_ Through a Peroxidase-Dependent Indirect Mechanism

**Figure 2:**
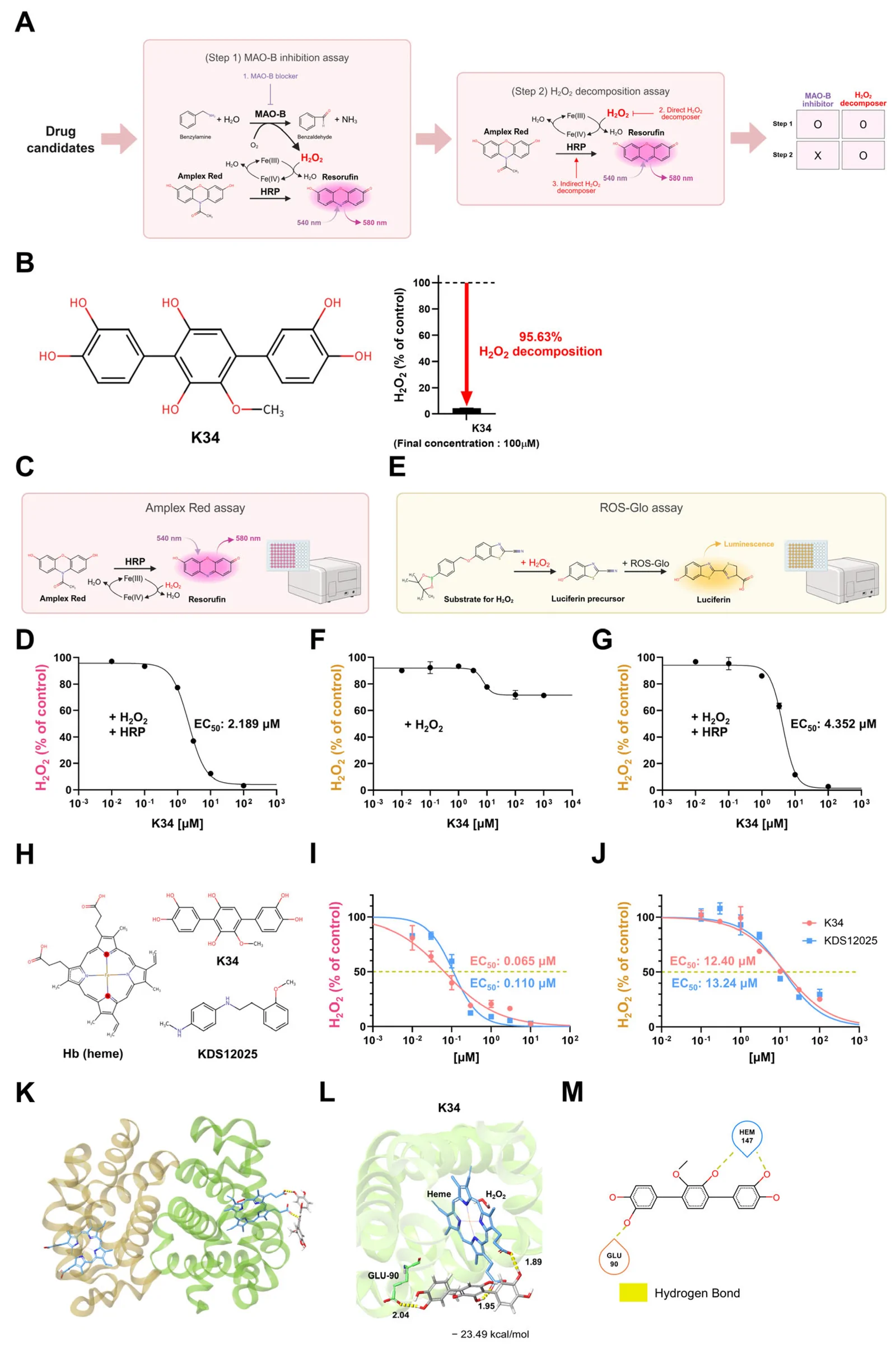
K34 promotes peroxidase-dependent H_2_O_2_ decomposition and is predicted to occupy the Hbβ heme pocket. (**A**) Schematic of the two-step screening strategy to identify peroxidase-dependent H_2_O_2_-decomposing compounds. (**B**) (left) Chemical structure of K34 (3,3″-dihydroxy-6′-desmethyl terphenyllin), isolated from the marine fungus *Penicillium janthinellum* 168 CLC-17.1, (right) and its H_2_O_2_-decomposing activity at a final concentration of 100 μM. The red downward arrow indicates the decrease in residual H_2_O_2_ following K34 treatment. (**C**) Schematic of the HRP-dependent Amplex Red H_2_O_2_ assay. (**D**) Dose–response curve for K34 in the Amplex Red assay. (**E**) Schematic of the HRP-independent ROS-Glo H_2_O_2_ assay. (**F**) Dose–response curve for K34 in the ROS-Glo assay in the absence of HRP. (**G**) Dose–response curve for K34 in the ROS-Glo assay in the presence of HRP. (**H**) Chemical structures of hemoglobin (Hb) heme, K34, and KDS12025, a structurally distinct synthetic Hb pseudoperoxidase enhancer based on an N-phenethylaniline scaffold. (**I**) Dose–response curves for K34 and KDS12025 for Hb-supported H_2_O_2_ decomposition in the Amplex Red assay. (**J**) Dose–response curves for K34 and KDS12025 for Hb-supported H_2_O_2_ decomposition in the ROS-Glo assay. (**K**) General view of the predicted K34 pose in the ferryl-state Hb model derived from PDB 2DN1 as proposed by docking simulations—Hbα, wheat; Hbβ, green. The heme group and K34 are shown as sticks. (**L**) Predicted binding modes of K34 (ΔGbind = −23.49 kcal/mol) within the Hbβ heme-binding pocket. Key residue interactions and distances between H_2_O_2_ and the ligand and protein (Å) are labeled. Binding energies were estimated via MM-GBSA. (**M**) Two-dimensional ligand interaction diagram of K34 binding to Hbβ generated via Schrödinger Maestro (ver. 14.2). Hydrogen bonds (yellow) are indicated. Dose–response curves and EC_50_ values were determined via GraphPad Prism (ver. 10.3.1) software. Data are presented as the means ± s.e.m. Panels (**A**,**C**,**E**) were partially created in BioRender. Lab, J. (2026) https://BioRender.com/j6o3rsc.

To identify H_2_O_2_-decomposing compounds from marine-derived small-molecule libraries, we developed a two-step screening strategy (Figure 2A). Step 1 employed a monoamine oxidase B (MAO-B) inhibition assay using Amplex red/HRP to exclude compounds that suppress H_2_O_2_ production upstream via MAO-B blockade. Compounds that passed Step 1 were subsequently assessed for H_2_O_2_-decomposing activity in the presence of H_2_O_2_ and in the absence of Benzylamine (MAO-B substrate) and the MAO-B enzyme in Step 2. Through this screen, K34 was identified as a Step 2-positive candidate. At a final concentration of 100 μM, K34 decomposed 95.63% of exogenous H_2_O_2_ (Figure 2B).

To characterize this peroxidase dependency, we employed two independent H_2_O_2_ detection platforms. In the Amplex Red assay, which measures HRP-catalyzed oxidation of Amplex Red to resorufin (Figure 2C), K34 dose-dependently reduced H_2_O_2_ in the presence of HRP with an EC_50_ of 2.189 μM (Figure 2D). In the ROS-Glo assay, which directly detects residual H_2_O_2_ via luciferin-based bioluminescence reaction independent of peroxidase activity (Figure 2E), K34 showed minimal H_2_O_2_-decomposing activity in the absence of HRP (Figure 2F) but exhibited dose-dependent activity upon HRP replenishment (EC_50_ = 4.352 μM; Figure 2G). The consistent HRP-dependent activity observed across two mechanistically distinct assay platforms demonstrates that K34 promotes peroxidase-dependent H_2_O_2_ decomposition.

### 3.3. K34 Promotes Hb-Supported H_2_O_2_ Decomposition and Is Predicted to Occupy the Hbβ Heme Pocket

Since hemoglobin (Hb) is a heme-containing protein expressed in astrocytes that exhibits pseudoperoxidase activity [4], we next examined whether Hb could support K34-dependent H_2_O_2_ decomposition in a purified-protein system (Figure 2H). In the Amplex Red assay with Hb substituted as the peroxidase, K34 dose-dependently decomposed H_2_O_2_ with an EC_50_ of 0.065 μM. Under the tested assay conditions, K34 displayed a lower apparent EC_50_ in the presence of Hb than HRP (Figure 2D,I). KDS12025, a synthetic small-molecule Hb pseudoperoxidase enhancer based on an N-phenethylaniline scaffold [4], showed a comparable EC_50_ of 0.110 μM (Figure 2I). In the ROS-Glo assay with Hb, EC_50_ values were 12.40 μM for K34 and 13.24 μM for KDS12025 (Figure 2J), with the quantitative difference between assays reflecting their mechanistic distinctions in H_2_O_2_ detection rather than a change in the relative potency of the two compounds. 

To investigate the structural basis of the K34-Hb interaction, we performed molecular docking simulations using Schrödinger Maestro. K34 docked within a wide cleft near the heme group of the Hbβ subunit (PDB: 2DN1; Figure 2K), with a binding free energy (ΔG_bind_) of −23.49 kcal/mol estimated by MM-GBSA (Figure 2L). Two-dimensional ligand interaction analysis revealed that the predicted pose placed K34 in hydrogen-bonding proximity to GLU-90 and the β-chain heme prosthetic group (HEM-147; residue identifier assigned to heme in PDB 2DN1) (Figure 2M). This *in silico* model provides a plausible structural hypothesis consistent with the Hb-supported H_2_O_2_-decomposing activity observed in the purified-protein assays.

### 3.4. K34 Reduces Aβ_42_-Induced Aberrant H_2_O_2_ in Primary Astrocytes

**Figure 3:**
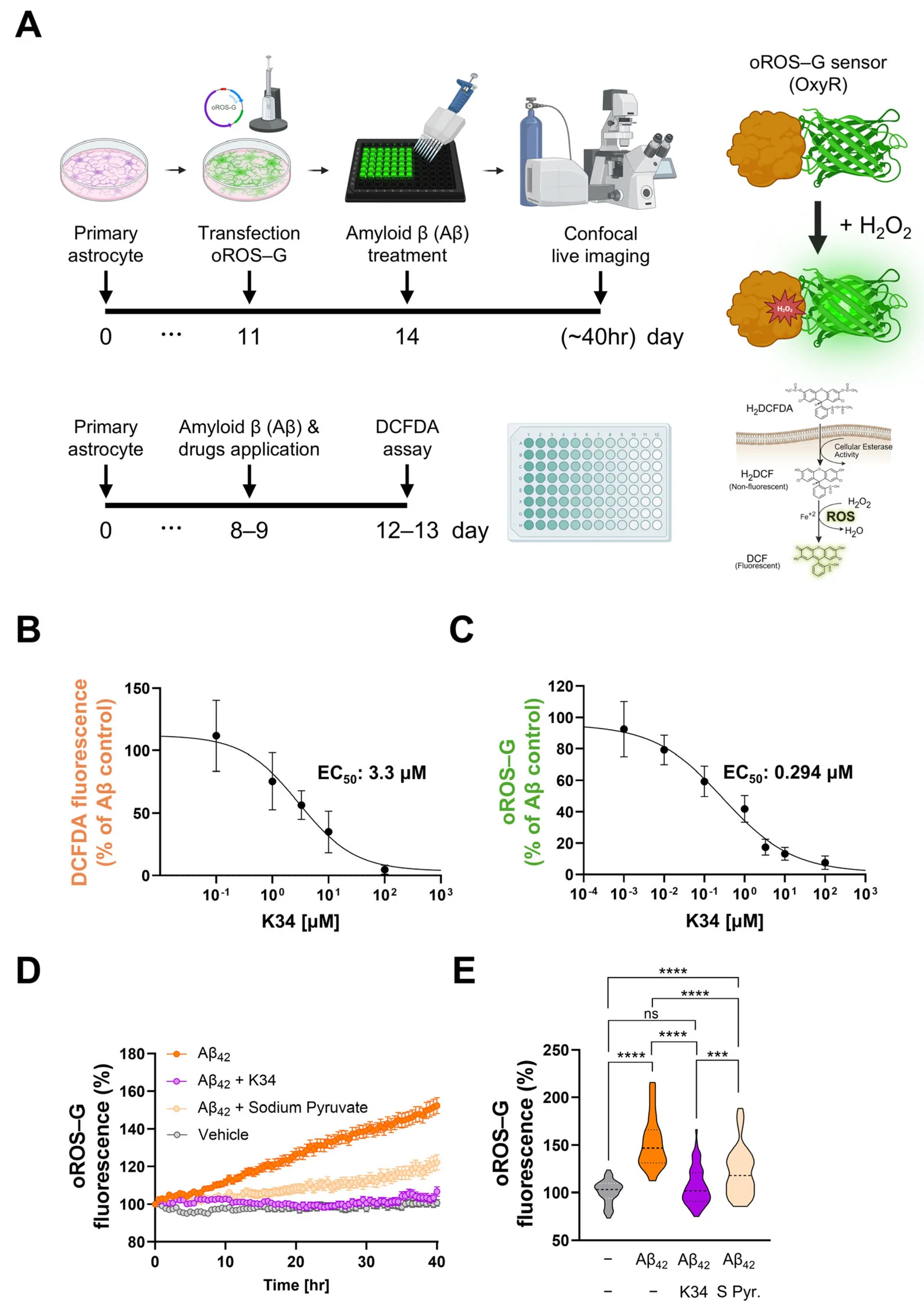
K34 reduces aberrant intracellular H_2_O_2_ in Aβ_42_-stimulated primary astrocytes. (**A**) Experimental timelines for H_2_O_2_ detection in primary cultured hippocampal astrocytes. (**B**) Dose–response curve for K34 in the DCFDA assay in Aβ_42_-treated astrocytes. (**C**) Dose–response curve for K34 in the oROS-G assay in Aβ_42_-treated astrocytes. (**D**) Continuous H_2_O_2_ imaging for 40 h via oROS-G probe following Aβ_42_ treatment, with administration of K34 and sodium pyruvate. Time-lapse traces of oROS-G fluorescence (%) for each condition are shown. (**E**) Endpoint quantification of oROS-G fluorescence following 40-h live imaging in (**D**) (n = 46, 40, 66, and 40 cells for vehicle, Aβ_42_, Aβ_42_+K34, and Aβ_42_+sodium pyruvate, respectively). Data are presented as the means ± s.e.m. for (**B**–**D**). Violin plots show the distribution of individual cells; the thick dashed line indicates the median and the thin dashed lines the 25th and 75th percentiles in (**E**). One-way ANOVA followed by Tukey’s multiple comparison test for (**E**). *** *p* < 0.001; **** *p* < 0.0001; ns, not significant. Panel A was partially created in BioRender. Lab, J. (2026) https://BioRender.com/j6o3rsc.

Having established that K34 decomposes H_2_O_2_ in a peroxidase-dependent manner in cell-free systems, we next examined whether it retains this activity in a physiologically relevant cellular context. We used primary cultured hippocampal astrocytes stimulated with oligomerized amyloid-β42 (Aβ_42_), which drives aberrant intracellular H_2_O_2_ production and models the reactive astrocyte-associated oxidative stress observed in AD [3]. Astrocytes have been reported to express hemoglobin [4,8], providing a biologically relevant cellular context in which to examine the H_2_O_2_-lowering effect of K34. We used two complementary detection methods in parallel (Figure 3A): the DCFDA assay for bulk intracellular reactive oxygen species (ROS) and the H_2_O_2_-specific genetically encoded sensor oROS-G for real-time live imaging.

In Aβ_42_-stimulated astrocytes, K34 dose-dependently reduced intracellular ROS with an EC_50_ of 3.3 μM in the DCFDA assay (Figure 3B) and with a lower EC_50_ of 0.294 μM in the oROS-G assay (Figure 3C). This difference reflects the distinct chemical selectivities of the two probes: DCFDA responds broadly to multiple reactive oxygen species, whereas oROS-G is selective for H_2_O_2_ [28,29]. Because DCFDA and oROS-G differ substantially in chemical selectivity and signal generation, their apparent EC_50_ values should not be compared quantitatively. Nevertheless, both assays showed reduced Aβ_42_-induced oxidative signals, while the H_2_O_2_-selective oROS-G sensor specifically demonstrated a reduction in intracellular H_2_O_2_. Forty-hour continuous live imaging showed that Aβ_42_ progressively elevated oROS-G fluorescence, and this increase was markedly attenuated by K34 (Figure 3D). Endpoint quantification confirmed that K34 significantly reduced Aβ_42_-induced H_2_O_2_. Compared to sodium pyruvate, a direct H_2_O_2_ scavenger [30], K34 reduced oROS-G fluorescence more strongly than sodium pyruvate under the tested conditions (Figure 3E). To assess whether the reduction in intracellular ROS/H_2_O_2_ signals was accompanied by changes in cellular viability-associated metabolic activity, we performed an additional WST-8 assay over 0.1–100 μM K34 (Figure S2). WST-8 activity showed comparatively modest reductions at sub- to low-micromolar K34 concentrations but declined more clearly at higher concentrations, particularly at 100 μM, indicating that altered viability-associated metabolic activity may contribute to the oxidative-signal reduction at higher concentrations. Together, these results demonstrate that K34 reduces Aβ_42_-induced intracellular H_2_O_2_ in primary astrocytes.

### 3.5. K34 Attenuates Memory Impairment, Reactive Astrogliosis, Neuronal Loss, and Hippocampal Circuit Dysfunction in the APP/PS1-Based fGiD AD Model

**Figure 4:**
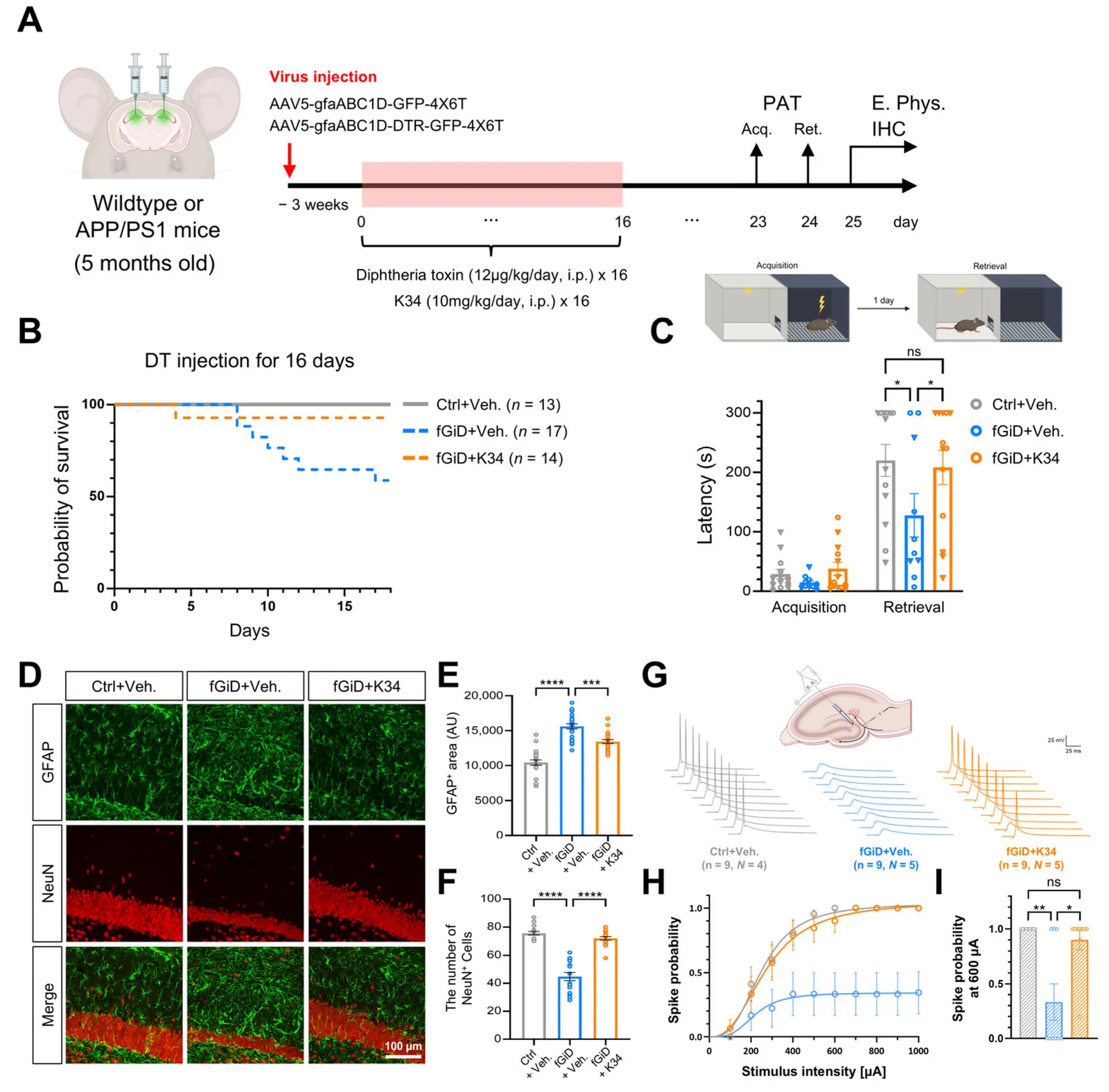
K34 attenuates memory impairment, reactive astrogliosis, neuronal loss, and hippocampal circuit dysfunction in the APP/PS1-based fGiD AD model. (**A**) Schematic timeline of the focal GiD (fGiD) AD model and K34 treatment. Wildtype or APP/PS1 mice (5 months old) received bilateral AAV injection (AAV5-gfaABC1D-GFP-4X6T and AAV5-gfaABC1D-DTR-GFP-4X6T) targeting astrocytes, followed by diphtheria toxin (DT; 12 μg/kg/day, i.p. × 16 days) and concurrent K34 (10 mg/kg/day, i.p. × 16 days). (**B**) Survival probability curves for Ctrl+Veh. (N = 13), fGiD+Veh. (N = 17), and fGiD+K34 (N = 14) groups over an 18-day observation period encompassing the 16-day course of DT injection (log-rank test; overall *p* = 0.0076; pairwise *p* values Bonferroni-corrected for 3 comparisons). (**C**) Step-through latency (s) in the PAT during acquisition and retrieval sessions for Ctrl+Veh. (N = 13), fGiD+Veh. (N = 10), and fGiD+K34 (N = 13) groups. Individual data points are color/shape-coded by sex (● male, ▼ female); Data were analyzed pooled across sex; exploratory within-group comparisons by sex were not significant (all *p* > 0.3) (**D**) Representative immunofluorescence images of the hippocampal CA1 region stained for GFAP and NeuN in Ctrl+Veh., fGiD+Veh., and fGiD+K34 mice (N = 3 per group [Ctrl+Veh., 2 female/1 male; fGiD+Veh., 2 female/1 male; fGiD+K34, 1 female/2 male]; scale bar, 100 μm). (**E**) Quantification of GFAP-positive area (AU) in hippocampal sections. (**F**) Quantification of NeuN-positive cell number in hippocampal sections. (**G**) Representative extracellular electrophysiology traces and schematic of spike probability measurements from hippocampal perforant pathway stimulation in Ctrl+Veh. (N = 4), fGiD+Veh. (N = 5), and fGiD+K34 mice (N = 5), (n = 9 recordings). (**H**) Spike probability across stimulation intensities (100–1000 μA) for Ctrl+Veh., fGiD+Veh., and fGiD+K34 groups. (**I**) Spike probability at 600 μA stimulus intensity for each group. Data are presented as the means ± s.e.m. One-way ANOVA followed Tukey’s multiple comparison test for (**C**,**E**,**F**). The Kruskal–Wallis test for (**I**). * *p* < 0.05, ** *p* < 0.01, *** *p* < 0.001; **** *p* < 0.0001; ns, not significant. Panels (**A**,**C**,**G**) were partially created in BioRender. Lab, J. (2026) https://BioRender.com/j6o3rsc.

To evaluate the in vivo neuroprotective effects of K34 in an AD-related neurodegenerative context, we employed the previously characterized APP/PS1-based focal GiD (fGiD) AD model, in which astrocyte-targeted DTR activation induces severe reactive astrogliosis and precipitates robust neurodegeneration and cognitive impairment [3] (Figure 4A). Wild-type or APP/PS1 mice (5 months old) received bilateral AAV injection targeting astrocytes, followed by DT administration (12 µg/kg/day, i.p. × 16 days) with concurrent K34 treatment (10 mg/kg/day, i.p. × 16 days).

Survival curves differed significantly among the three groups overall (*p* = 0.0076); however, K34 did not significantly improve survival compared with fGiD+Veh. after correction for multiple comparisons (*p* = 0.132) (Figure 4B). Cox proportional hazards regression showed no significant main effect of sex on survival (hazard ratio (HR) = 0.43, *p* = 0.25). Because no fGiD+K34-treated male died during the observation period, the treatment × sex interaction term could not be reliably estimated (log-rank, female *p* = 0.075; male *p* = 0.195). In the passive avoidance test (PAT), fGiD+Veh. mice showed substantially reduced step-through latency at retrieval compared to Ctrl+Veh. mice, reflecting hippocampus-dependent memory impairment, and this deficit was significantly attenuated by K34 treatment (Figure 4C). No significant differences were observed among groups at acquisition, confirming comparable baseline behavior before the memory encoding trial.

Immunofluorescence analysis of hippocampal sections revealed a markedly elevated GFAP-positive area in fGiD+Veh. mice compared to Ctrl+Veh. mice, indicative of severe reactive astrogliosis, and this was significantly reduced by K34 treatment (Figure 4D,E). Concurrently, the number of NeuN-positive neurons was drastically reduced in fGiD+Veh. mice, and K34 treatment significantly preserved neuronal survival towards control levels (Figure 4F).

To assess hippocampal circuit function, we performed extracellular electrophysiology via perforant pathway stimulation (Figure 4G). Spike probability across stimulus intensities (100–1000 µA) was markedly reduced in fGiD+Veh. mice compared to Ctrl+Veh. mice, reflecting impaired neural circuit excitability (Figure 4H). At 600 µA stimulus intensity, fGiD+Veh. mice showed significantly lower spike probability than Ctrl+Veh. mice, and K34 treatment significantly improved spike probability, with no significant difference remaining between fGiD+K34 and Ctrl+Veh. groups (Figure 4I). Together, these results show that K34 attenuated memory impairment and reactive astrogliosis, preserved NeuN-positive neurons, and improved hippocampal spike probability in the APP/PS1-based fGiD AD model.

### 3.6. K34 Exhibits Low Passive BBB Permeability and Limited CYP Inhibition In Vitro 

Following the in vivo evaluation of K34, we next evaluated its selected in vitro developability-related properties. To this end, we characterized its blood–brain barrier (BBB) permeability and cytochrome P450 (CYP) inhibition profile in vitro (Table 1). In a parallel artificial membrane permeability assay (PAMPA-BBB), K34 exhibited an effective permeability (P_e_) of 0.17 × 10^−^^6^ cm/s, well below the threshold for high BBB penetration, and was accordingly classified as CNS− (low BBB permeability); the reference compounds progesterone (CNS+) and ranitidine (CNS−) validated the assay. In parallel, K34 showed no meaningful inhibition of the five major human CYP isozymes (CYP1A2, CYP2C9, CYP2C19, CYP2D6, and CYP3A4), with all IC_50_ values exceeding 10 μM, suggesting limited inhibition of these five CYP isoforms within the tested concentration range. Together, these results indicate that K34 has limited passive BBB permeability, a property profile we consider further in the context of its proposed mechanism of action below.

## 4. Discussion

In this study, we isolated the catechol-bearing para-terphenyl K34 from the marine-derived fungus *Penicillium janthinellum* 168CLC-17.1 and identified K34 as a natural product that promotes peroxidase-dependent H_2_O_2_ decomposition (Figure 1). Using complementary cell-free assays, we find that K34 promotes peroxidase-dependent H_2_O_2_ decomposition and Hb-supported H_2_O_2_ decomposition, while molecular docking predicts a plausible binding pose near the Hbβ heme pocket (Figure 2). In Aβ-stimulated primary astrocytes, K34 reduced aberrant intracellular H_2_O_2_ with sub-μM potency (Figure 3), and in the APP/PS1-based fGiD AD model, systemic administration of K34 attenuated memory impairment and reactive astrogliosis, preserved NeuN-positive neurons, and improved hippocampal circuit function (Figure 4). Together, these findings identify K34 as a terphenyllin-type natural product that promotes Hb-supported H_2_O_2_ decomposition in purified-protein assays and attenuates selected neurodegenerative phenotypes in the APP/PS1-based fGiD AD model.

The mechanism we describe differs fundamentally from that of conventional direct antioxidants. Agents such as sodium pyruvate, N-acetylcysteine, and Vitamin C consume H_2_O_2_ stoichiometrically and therefore require high concentrations in the mM range to be effective [6,31,32], often with nonspecific reactivity. In contrast, K34 promotes Hb-supported H_2_O_2_ decomposition through a peroxidase-dependent mechanism, with activity observed at substantially lower concentrations than those required for direct scavengers such as sodium pyruvate. Strikingly, K34 shares this potent functional property with our previously reported synthetic Hb pseudoperoxidase enhancers, including KDS12025, despite being structurally unrelated to them: whereas those compounds, including KDS12025, are built on an N-phenethylaniline scaffold bearing a proton- and electron-donating amine group [4], K34 is a polyhydroxylated para-terphenyl that lacks this amine entirely, instead possessing two catechol moieties on both sides (Figure 2H). These catechol moieties of K34 are well-established proton and electron donors [33] and could therefore provide reducing equivalents during the Hb–H_2_O_2_ reaction. The observation that two structurally distinct chemotypes promote Hb-supported H_2_O_2_ decomposition suggests that this activity can be achieved by chemically distinct scaffolds (Figure 2K–M). This mechanistic profile also provides a useful point of comparison with better-studied catechol-containing natural polyphenols, including EGCG, quercetin, and piceatannol. These compounds have shown neuroprotective effects in AD-related experimental systems and have been associated with multiple mechanisms, including antioxidant, anti-inflammatory, and Aβ-related activities [18,19,20,21]. K34 shares with these compounds a redox-active polyphenolic architecture and the capacity to reduce oxidative stress-associated signals but differs in that its H_2_O_2_-decomposing activity in the present cell-free assays was strongly peroxidase-dependent and was supported by Hb. Thus, the principal distinction of K34 is mechanistic rather than evidence of greater antioxidant or therapeutic efficacy. Because no direct head-to-head comparison was carried out, relative potency or efficacy among these compounds cannot be inferred from the present study.

Molecular docking provided a structural hypothesis consistent with this interpretation. In the predicted pose, K34 was positioned within the Hbβ heme pocket rather than at the surface cleft occupied by the synthetic enhancers. In the predicted pose, one catechol hydroxyl hydrogen-bonded to the heme cofactor (1.89 Å) while the opposing catechol hydroxyl was anchored by Glu90 (2.04 Å) (Figure 2M), positioning a catechol ring adjacent to the heme iron in a geometry compatible with hydrogen-atom delivery to the bound H_2_O_2_. This predicted binding pose is compatible with a proposed reaction model in which the heme-proximal catechol could provide reducing equivalents during the Hb pseudoperoxidase reaction (Figure S3). In the Hb pseudoperoxidase reaction, H_2_O_2_ coordinates to the heme iron, and its O–O bond is cleaved with release of water, a process that requires an electron- and proton-donating partner to stabilize the developing intermediates [10,11]. The synthetic enhancers of this class provide this donor through their electron-donating aniline N–H group, which has been proposed to mimic a missing polar residue and to facilitate proton and electron transfer at the heme [4]. K34 lacks this amine but instead carries two catechol (3,4-dihydroxyphenyl) moieties on its outer terphenyl rings. Catechols are well-characterized hydrogen-atom donors: loss of the first phenolic hydrogen yields a semiquinone radical that is stabilized both by an intramolecular hydrogen bond to the adjacent hydroxyl and by the electron-donating character of the ortho-hydroxyl, lowering the effective O–H bond dissociation enthalpy, after which the semiquinone can donate a second hydrogen to form the corresponding ortho-quinone [34]. In this hypothetical model, the heme-proximal catechol of K34 could engage the iron-bound H_2_O_2_ through a hydrogen-bond network and donate a catechol-derived hydrogen equivalent, potentially facilitating O–O bond cleavage and water release while forming a semiquinone-like intermediate (Figure S3). Such a reaction pathway could provide a chemically distinct mode of redox assistance compared with the amine-containing KDS12025; however, the redox fate and regeneration of K34 were not directly determined.

Because both the enhanced Hb–H_2_O_2_ reaction and the catechol chemistry invoked above could in principle generate reactive species—ferryl-Hb and secondary protein radicals on the enzyme side [10], and semiquinone radicals or electrophilic ortho-quinones on the ligand side [34]—the possibility of a prooxidant shift warrants consideration. Several observations argue against this under the conditions examined: K34 produced a net reduction in H_2_O_2_ in a H_2_O_2_-selective cellular sensor and improved, rather than worsened, neuronal and behavioral outcomes in vivo. Moreover, the pronounced hydrophilicity of K34, possibly reflected in its low membrane permeability, is a physicochemical feature generally associated with a lower propensity for quinone-mediated cytotoxicity among catecholic compounds [34]. A detailed characterization of K34 oxidation products and radical/quinone species formed during the Hb–H_2_O_2_ reaction will be required to test this proposed mechanism.

An apparent discrepancy in the cell-free data warrants clarification. Although both cell-free assays used Hb as the peroxidase enzyme, the EC_50_ values differed by approximately two orders of magnitude (~0.1 μM by Amplex Red versus ~13 μM by ROS-Glo, Figure 2I,J). The approximately two-order-of-magnitude difference in apparent EC_50_ values should not be interpreted as a direct difference in intrinsic potency, because the two assays use distinct detection principles and dynamic ranges. Amplex Red is itself a peroxidase-coupled reporter and is therefore particularly sensitive to changes in peroxidase-mediated reporter turnover, whereas ROS-Glo measures residual H_2_O_2_ through an orthogonal detection chemistry [35,36]. Comparable assay-dependent shifts have been noted for structurally related peroxidase enhancers [4], and the two readouts should thus be interpreted as complementary rather than contradictory.

A central consideration for the translational interpretation of these findings is the limited BBB permeability of K34 (Table 1; P_e_ = 0.17 × 10^−^^6^ cm/s, CNS−), together with its favorable peripheral profile (no meaningful CYP inhibition; IC_50_ > 10 μM, Table 1). This property also distinguishes K34 from the BBB-permeable synthetic enhancers of this class, whose therapeutic action in the AD brain has been attributed primarily to central, astrocytic Hb [4]. The low PAMPA-BBB permeability of K34 suggests limited passive BBB diffusion. Together with its phenotypic effect after systemic administration, this observation raises the possibility that peripheral mechanisms contribute to its in vivo effects. One hypothetical mechanism is K34-facilitated H_2_O_2_ decomposition by circulating Hb, which could alter the peripheral–central H_2_O_2_ gradient. Because Hb is abundant in circulating erythrocytes [7], the circulation represents a plausible peripheral site at which systemically administered K34 could act; however, this possibility was not directly tested in the present study. Given that H_2_O_2_ is membrane-permeant and can diffuse across the BBB along its concentration gradient [37,38], sustained decomposition of circulating H_2_O_2_ could lower peripheral H_2_O_2_ and promote net efflux of brain-derived H_2_O_2_ into the blood, indirectly reducing the central oxidative burden. This model is supported by prior demonstrations that a synthetic enhancer of this class reduced lipopolysaccharide (LPS)-elevated blood H_2_O_2_ and that purified erythrocytes decompose H_2_O_2_ in a compound-dependent manner [4]. Notably, oxidative damage markers are elevated not only in plasma but also in circulating erythrocytes of AD patients [39,40], consistent with an oxidative burden in the very compartment where K34 is proposed to act.

Our cellular experiments were designed to test whether K34 retains H_2_O_2_-decomposing activity in a relevant biological setting, rather than to define its site of action in vivo. Primary astrocytes were chosen because they generate Aβ-induced H_2_O_2_ and have been reported to express Hb, providing an Hb-expressing cellular context relevant to the mechanism identified in purified-protein assays [4,8]. These experiments demonstrate that K34 lowers Aβ-induced H_2_O_2_ in primary astrocytes; however, they do not establish that this effect depends on astrocytic Hb, and contributions from other redox mechanisms cannot be excluded. The additional WST-8 analysis showed comparatively modest changes in viability-associated metabolic activity at sub- to low-micromolar K34 concentrations, whereas a more pronounced decrease was observed at higher concentrations, particularly at 100 μM (Figure S2). Because reductions in intracellular ROS/H_2_O_2_ were already observed within the concentration range where changes in WST-8 activity were comparatively modest, these effects are unlikely to be explained solely by reduced cellular viability or metabolic activity; however, such changes may contribute to the effects observed at higher concentrations. With respect to the possible in vivo site of action, brain Hb is low in abundance relative to the circulation and is further depleted in the AD brain [4], reinforcing the plausibility of a peripheral locus; at the same time, enhancers of this class can drive H_2_O_2_ decomposition even at very low Hb concentrations, so a minor central contribution cannot be excluded.

Several limitations should be acknowledged. First, although purified-protein assays support Hb in K34-associated H_2_O_2_ decomposition, Hb dependence was not directly tested in primary astrocytes or in vivo; therefore, the cellular and neuroprotective effects of K34 cannot yet be attributed specifically to Hb pseudoperoxidase enhancement. Second, even though our systemic equilibrium-shift model is supported by the peripheral activity of related compounds and by the biophysical properties of H_2_O_2_, we did not directly measure circulating or brain H_2_O_2_ in K34-treated animals; this model therefore remains to be tested experimentally. Third, only a single systemic dose of K34 was evaluated, and pharmacokinetic measurements were not performed; therefore, the dose–response relationship and the systemic or brain exposure achieved at 10 mg/kg/day remain unresolved. In addition, the PAMPA assay measures only passive transcellular diffusion and does not exclude active transport or alternative routes of brain entry. Fourth, although the APP/PS1-based fGiD model has been previously characterized as an AD model with pronounced neurodegenerative phenotypes [3], the present study did not directly assess whether K34 modifies canonical Aβ or tau pathology. Therefore, the in vivo effects of K34 should be interpreted in terms of the behavioral, histological, and electrophysiological phenotypes examined here, and validation in additional AD models will be required to establish their broader generalizability. Finally, the study was not powered to detect sex-specific differences in treatment efficacy; in particular, the absence of any death among fGiD+K34-treated males precluded a stable statistical estimate of the treatment × sex interaction.

## 5. Conclusions

K34 is a marine-derived para-terphenyl that promotes peroxidase-dependent H_2_O_2_ decomposition in cell-free assays, including Hb-supported H_2_O_2_ decomposition, and reduces Aβ_42_-associated intracellular H_2_O_2_ in primary astrocytes. In APP/PS1 mice subjected to focal GiD, systemic K34 attenuated memory impairment and reactive astrogliosis, preserved NeuN-positive neurons, and improved hippocampal circuit function. Together, these findings identify K34 as a natural-product scaffold for indirect H_2_O_2_ regulation, while the contribution of Hb to its cellular and in vivo effects, its site of action, and its relevance to canonical AD pathology remain to be established.

## Figures and Tables

**Table 1:** In vitro BBB permeability and CYP inhibition profile of K34.

Compound	pH	P_e_ (10^−6^ cm/s) Avg ± SD		BBB Permeability	
PAMPA-BBB Permeability					
K34 (50 μM)	7.4	0.17 ± 0.01		CNS−	
Progesterone (*ref*)	7.4	28.84 ± 0.42		CNS+	
Ranitidine (*ref*)	7.4	0		CNS−	
**Compound**	**CYP1A2**	**CYP2C9**	**CYP2C19**	**CYP2D6**	**CYP3A4**
CYP Inhibition IC_50_ (μM)					
K34	>10	>10	>10	>10	>10

P_e_, effective permeability (mean ± SD, n = 3). CNS+, high BBB permeability (P_e_ > 10 × 10^−^^6^ cm/s); CNS−, low BBB permeability. CYP inhibition classification: potent, IC_50_ < 1 μM; moderate, 1–10 μM; no/weak inhibition, IC_50_ > 10 μM. *ref*, reference compound.

## Data Availability

The raw data supporting the conclusions of this article will be made available by the authors upon reasonable request.

## Supplementary Materials

The following supporting information can be downloaded at https://www.mdpi.com/article/10.3390/antiox15101293/s1, Figure S1: Spectroscopic data for the structural elucidation of K34; Figure S2: Assessment of primary astrocyte viability-associated metabolic activity following K34 treatment using the WST-8 assay. Figure S3: Proposed reaction model for K34-facilitated Hb-associated H_2_O_2_ decomposition.

## Abbreviations

The following abbreviations are used in this manuscript:

AD	Alzheimer’s disease
Aβ_42_	amyloid-β42
AAV	adeno-associated virus
aCSF	artificial cerebrospinal fluid
ANOVA	analysis of variance
BBB	blood–brain barrier
CYP	cytochrome P450
DCFDA	2′,7′-dichlorodihydrofluorescein diacetate
DMSO	dimethyl sulfoxide
DT	diphtheria toxin
EC_50_	half-maximal effective concentration
EPSP	excitatory postsynaptic potential
ESIMS	electrospray ionization mass spectrometry
fGiD	focal GFAP-inducible diphtheria toxin receptor
GFAP	glial fibrillary acidic protein
Hb	hemoglobin
HMBC	heteronuclear multiple bond correlation
HPLC	high-performance liquid chromatography
HRP	horseradish peroxidase
HSQC	heteronuclear single quantum coherence
LC–MS/MS	liquid chromatography–tandem mass spectrometry
MAO-B	monoamine oxidase B
MM-GBSA	molecular mechanics/generalized Born surface area
NeuN	neuronal nuclei
NMR	nuclear magnetic resonance
PAMPA-BBB	parallel artificial membrane permeability assay for the blood–brain barrier
PAT	passive avoidance test
PBS	phosphate-buffered saline
PFA	paraformaldehyde
ROS	reactive oxygen species
