# Supplementary material for: K34 (3,3″-Dihydroxy-6′-desmethyl Terphenyllin) Promotes Hemoglobin-Supported H2O2 Decomposition and Attenuates Neurodegenerative Phenotypes in APP/PS1 Mice with Focal GiD
Source: Antioxidants (Basel). 2026 Oct 5;15(10):1293. doi: 10.3390/antiox15101293 (PMC13700790; doi:10.3390/antiox15101293)
Supplement: Supplementary file 1 [file antioxidants-15-01293-s001.zip › antioxidants-4529403-supplementary.pdf]

Supplementary Figures

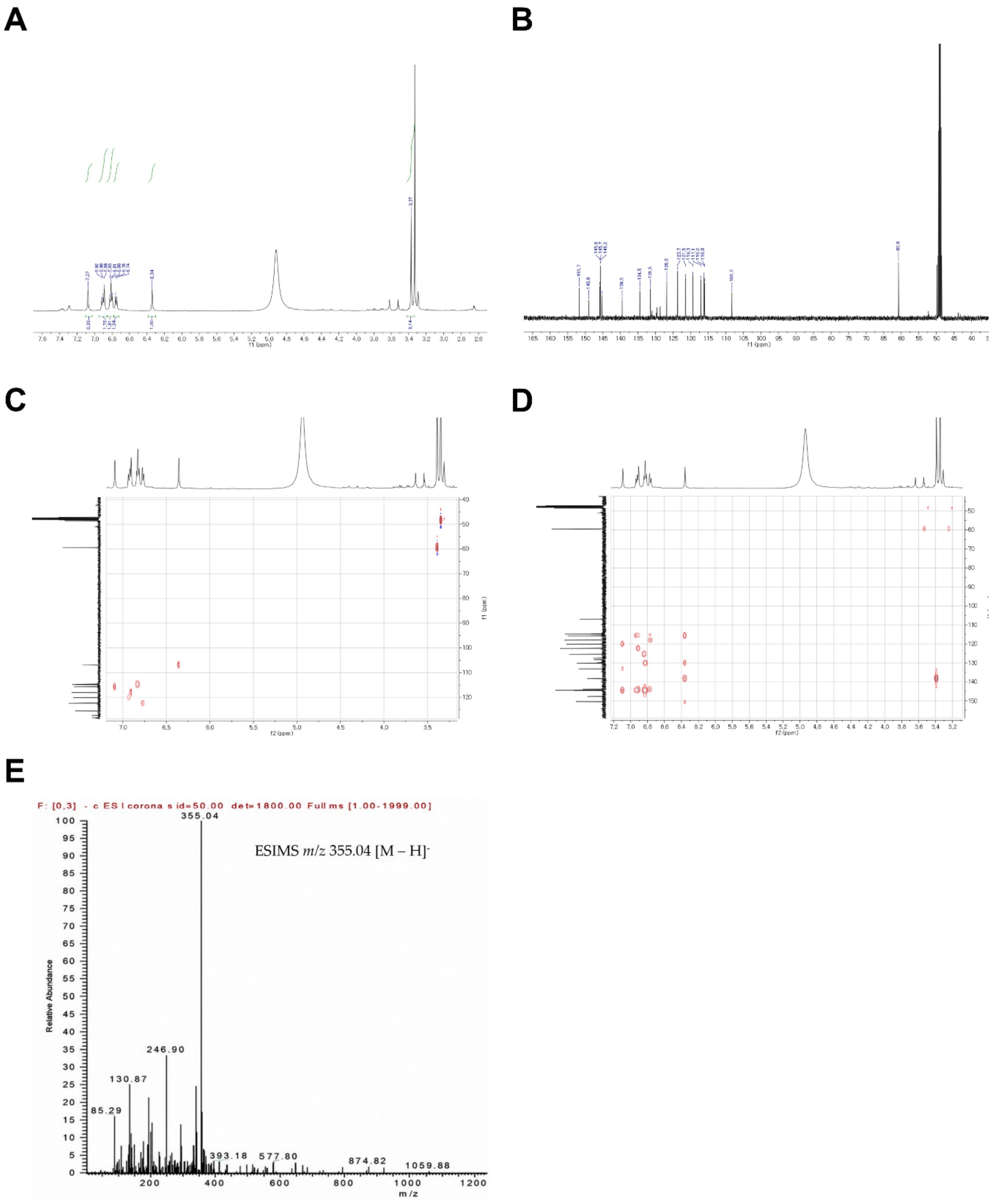

Figure S1 Spectroscopic data for the structural elucidation of K34. (A) ^{1}H NMR spectrum of K34. (B) ^{13}C NMR spectrum of K34. (C) HSQC spectrum of K34. (D) HMBC spectrum of K34. (E) Negative-ion ESIMS spectrum of K34.

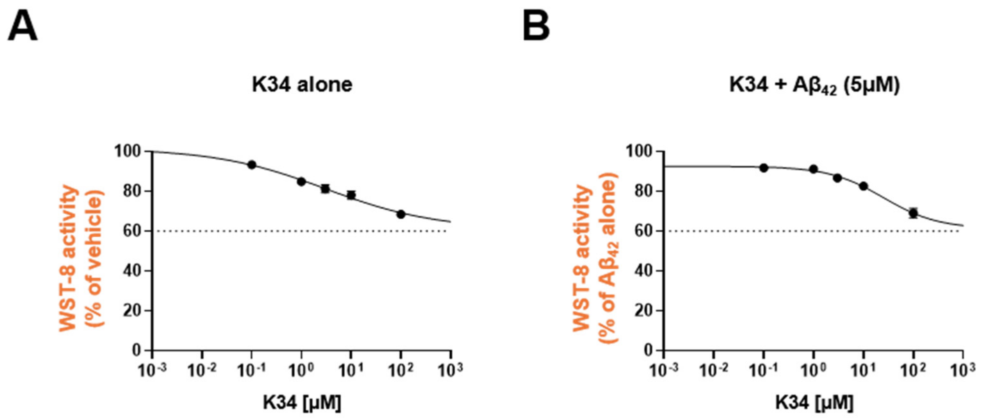

Figure S2 Assessment of primary astrocyte viability-associated metabolic activity following K34 treatment using the WST-8 assay. Primary astrocytes were treated with the indicated concentrations of K34 in the absence (A) or presence (B) of oligomerized $A\beta_{42}$ (5 µM) under conditions corresponding to the intracellular ROS/H_2O_2 experiments. WST-8 activity was normalized to vehicle in (A) and $A\beta_{42}$ alone in (B).

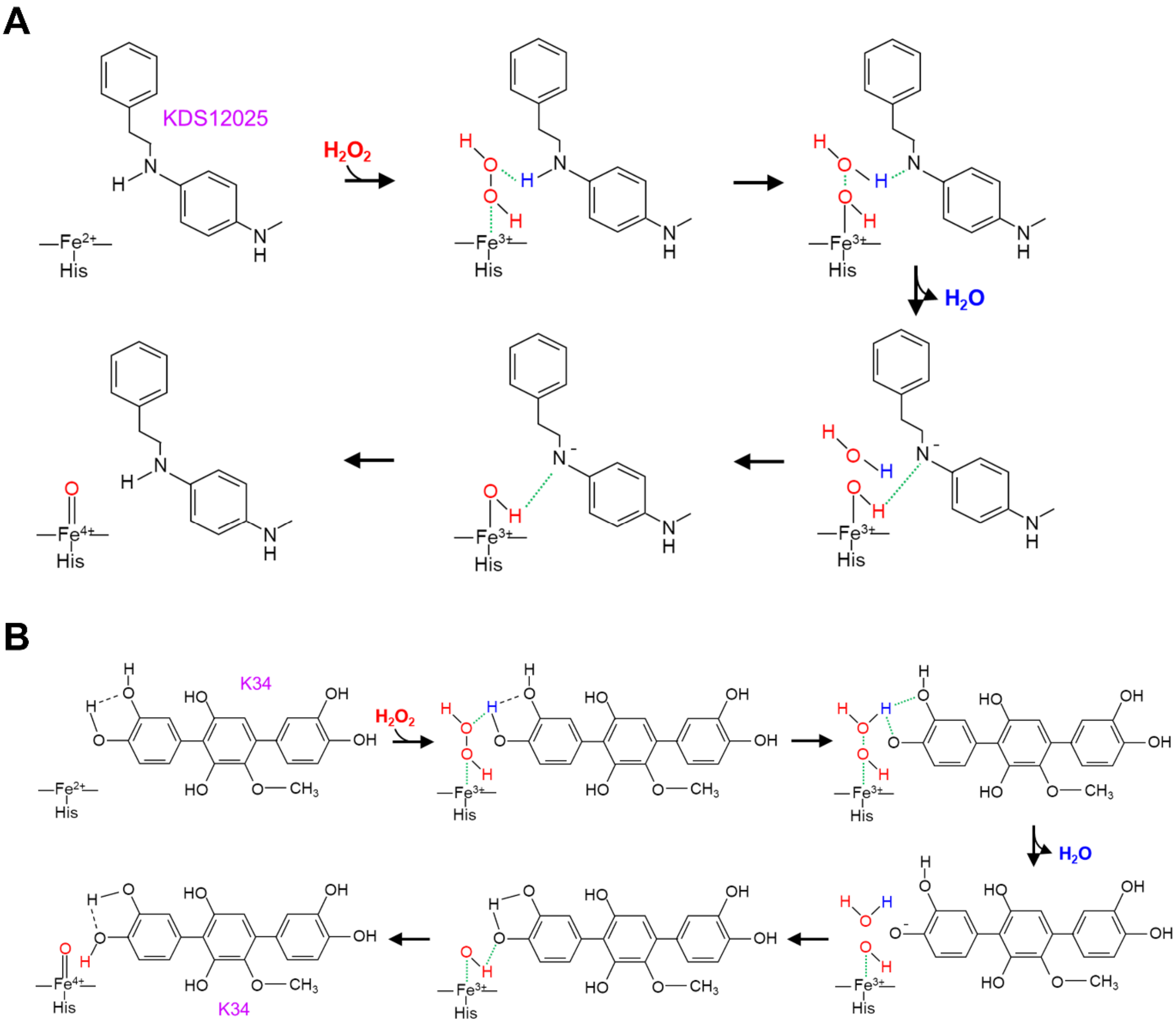

Figure S3 Proposed reaction model for K34-facilitated Hb-associated H_2O_2 decomposition. (A) Proposed reactive conformation model for the decomposition of H_2O_2 (red color) for proton transfer and release of H_2O (blue color) with KDS12025 assisting in coordinating H_2O and subsequently H_2O_2 with the iron atom. (B) Proposed reactive conformation model for the decomposition of H_2O_2 (red color) for proton transfer and release of H_2O (blue color) with K34 assisting in coordinating H_2O and subsequently H_2O_2 with the iron atom.
